# Maximal isometric and eccentric hamstring strength is influenced by body mass and additional load: Does the critical point at which peak knee flexor force is achieved play a role?

**DOI:** 10.3389/fphys.2025.1654030

**Published:** 2025-09-18

**Authors:** Andrew Rinaldi Sinulingga, Erika Zemková

**Affiliations:** ^1^ Department of Biological and Medical Sciences, Faculty of Physical Education and Sports, Comenius University in Bratislava, Bratislava, Slovakia; ^2^ Department of Anatomy, Physiology, Biochemistry, Biomchanics, Hygiene and Informatic, Riga Stradins University Latvian Academy of Sports Education, Riga, Latvia; ^3^ Department of Sport Education, University of Riau, Pekanbaru, Indonesia

**Keywords:** eccentric contraction, knee flexor strength, nordic hamstring exercise, peak force, isometric contraction

## Abstract

Isometric (ISO) and eccentric (ECC) hamstring tests are used to assess peak force at various knee angles. However, it is unknown to what extent body weight influences the so-called “critical point” at which individuals achieve their maximal knee flexor force production. This study compared (1) the peak force during maximum voluntary isometric contraction at 60^o^ knee flexion without body weight, with body weight and with added weight, and (2) the eccentric knee flexor strength during Nordic hamstring exercise (NHE) up to 60^o^ of knee flexion with body weight and added weight, as well as up to an angle of 20^o^ with body weight only. The relationship between peak isometric strength in all loading conditions and eccentric strength during NHE performed to different knee angles was also investigated. 22 male athletes (age 21.7 ± 4.3 years, height 181.6 ± 7.5 cm, body mass 75.4 ± 8.5 kg) completed i) maximal efforts on isometric knee flexion at 60° (ISO60), with body weight (ISO60-BW), and with 5 kg medicine ball (ISO60-BW + AW), ii) NHE with lean forward from 90^o^ to 60° with body weight (ECC60-BW) and a 5 kg medicine ball (ECC60-BW + AW), and iii) NHE with lean forward up to 20^o^ with body weight (ECC20). Results showed higher peak force during ISO60-BW + AW compared to ISO60-BW (24.2 N, 6.5%, p = 0.012), and ISO60 (42.1 N, 11.6%, p = 0.000). The added 5 kg (ECC60-BW + AW) produced greater eccentric force compared to body weight (ECC60-BW) (17.9 N, 6.4%; p = 0.03). However, there was no significant difference between NHE with body weight and added weight, irrespective of the subject’s ability to achieve a final position of 20^o^ of knee flexion. Relative eccentric force was higher in participants who achieved 20° than in those who reached their critical point at 45° of knee flexion (p = 0.001, d = 1.89). Peak isometric force in all three conditions significantly correlated with NHE peak force at 45° (r = 0.79–0.90) and 20° of knee flexion (r = 0.71–0.77), explaining 62%–81% and 49%–58% of the variance, respectively. These findings indicate that isometric and eccentric measures of hamstring strength are interdependent. However, eccentric hamstring strength during NHE is more dependent on maximal hamstring strength when this exercise is performed to a critical point of 45° than 20° of knee flexion.

## Introduction

The architecture of the hamstring muscles exhibits distinct structural and functional characteristics, each playing an important role in various movements of the lower extremities. The main functions of the hamstrings are knee flexion and hip extension. The long head (BFlh) and short head (BFsh) of the biceps femoris contribute to lateral rotation of the tibia, while semitendinosus (ST) and semimembranosus (SM) assist in medial rotation of the tibia (Rodgers and Raja, 2023; Takeda et al., 2023). The hamstring muscles play a role in human movement, supporting activities such as sprinting and jumping (Chumanov et al., 2007; Rodgers and Raja, 2023), transitioning from a seated to a standing position (Hanawa et al., 2017), and walking (Arnold et al., 2005). Improving hamstring strength has a significant impact on sprinting and jumping performance (Markovic et al., 2020; Váczi et al., 2022), as well as maximum speeds and running distances in professional soccer players (Jiménez-Rubio et al., 2019). Conversely, hamstring weakness is associated with an increased risk of hamstring strain injuries (HSI) (Petersen and Hölmich, 2005), which remain a significant concern due to their persistent symptoms, slow healing process, and high rate of re-injury. After HSI, deficits in isometric strength and passive straight leg raising typically recover within 20–50 days, while deficits in eccentric and concentric strength may persist after returning to play (Maniar et al., 2016).

Investigating isometric (ISO) and eccentric (ECC) hamstring strength can improve understanding of a subject’s ability to produce peak force and torque during different muscle contractions. A substantial body of research has explored the assessment of isometrics to understand the rate of force development at different angles of the knee flexor (Read et al., 2019; Taylor et al., 2025) to provide information on the risk of HSI given the proposed higher muscle activation of BFlh (Reurink et al., 2016). On the other hand, eccentric strength testing provides information for detecting, quantifying, and addressing strength deficits in patients and athletes (Lodge et al., 2020). Evidence has shown that low levels of eccentric hamstring strength increase the risk of future HSI (Opar et al., 2015). Therefore, isometric and eccentric hamstrings tests provide different information for assessing hamstring performance and rehabilitation (Moreno-Pérez et al., 2020).

Recent studies have revealed several important findings, including the influence of foot and body position on peak torque during hamstring strength tests (Claudino et al., 2021; Ogborn et al., 2021), and the validity and reliability of a novel device to measure eccentric/isometric knee flexion and extension (Opar et al., 2013; Toonstra and Mattacola, 2013; Hirano et al., 2020). Additionally, prior studies have investigated the effect of a fatigue protocol on peak force and rate of force development, as well as hamstring active muscle stiffness (Evangelidis et al., 2022; Bettariga et al., 2023). More closely related to the present study, Amundsen et al. (2023) investigated a Nordic hamstring strength test with added weight, which was shown to producee higher eccentric force. Similarly, Mjølsnes et al. (2004) proposed a progressive loading approach during the NHE based on the participant’s capacity to resist forward falling throughout the range of movement. This study revealed that the implementation of load progression within 10 weeks of NHE, participants gained 11% higher force compared to traditional hamstring curl exercise (Mjølsnes et al., 2004).

However, it is unknown to what extent body weight plus added weight influences isometric and eccentric hamstring strength and the so-called “critical point” at which individuals achieve their maximal knee flexor force production. It is the point where the increasing external load of gravity acting on the upper body exceeds the individual’s maximum eccentric hamstring strength. Our study aimed to investigate the effect of body weight and additional weight on isometric and eccentric hamstring strength. First, the peak force during maximum voluntary isometric contraction was compared for (1) knee flexion at 60^o^, (2) knee flexion at 60^o^ with body weight, and (3) knee flexion at 60^o^ with added weight of 5 kg. Second, the eccentric knee flexor strength was assessed during (1) NHE with lean forward up to 60^o^ knee flexion with body weight and added weight of 5 kg, and (2) up to an angle of 20^o^ with body weight only. In addition, the relationship between peak isometric force in all loading conditions and eccentric strength during NHE performed at different knee angles was examined. We hypothesized that 1) ISO60 with additional weight of 5 kg produces higher peak force than ISO60 with body weight and without it; 2) NHE performed to 60^o^ knee flexion produces higher peak force with added load compared to body weight; and 3) higher relative force is produced during NHE performed to 20^o^ than 45^o^ of knee flexion. We also assume that peak isometric strength is related to peak eccentric strength, but this relationship depends on the knee angle during NHE.

## Methods

### Participants

The present study had a cross-sectional design to examine the effect of body weight and additional weight on isometric and eccentric hamstring strength. A total of 22 male athletes (age 21.65 ± 4.30 years, height 181.55 ± 7.50 cm, body mass 75.44 ± 8.52 kg) volunteered to participate in the study. *A priori* power analysis was conducted using G*Power 1.9.4 to estimate the required sample size. The effect size (f) greater than 0.50, 80% statistical power (β = 0.8), and a significance level of α = 0.05, the minimum required sample size was calculated to be 19 participants, based on the experimental study design by Bettariga et al. (2023). The participants were enrolled in the team sports, athletics, combat sports, and multi-sports disciplines. We excluded participants with any history of lower extremity injuries or back pain. All subjects provided written informed consent prior to testing. This research was in accordance with the ethical standards on human experimentation conducted in compliance with the 1964 Helsinki Declaration and its subsequent modifications. This project was approved by the ethics committee of the Faculty of Physical Education and Sports, Comenius University in Bratislava (No. 2/2023).

### Isometric and eccentric hamstring strength testing

Maximal isometric and eccentric hamstring strength was measured using the FiTRO Hamstring Diagnostic System (FiTRONiC, Bratislava, Slovakia). Participants knelt on the padded board with their ankles secure close to the lateral malleolus by individual ankle braces. The examiner determined the lower leg lever of the athlete (from the knee-joint axis of rotation to the ankle strap).

**FIGURE 1:**
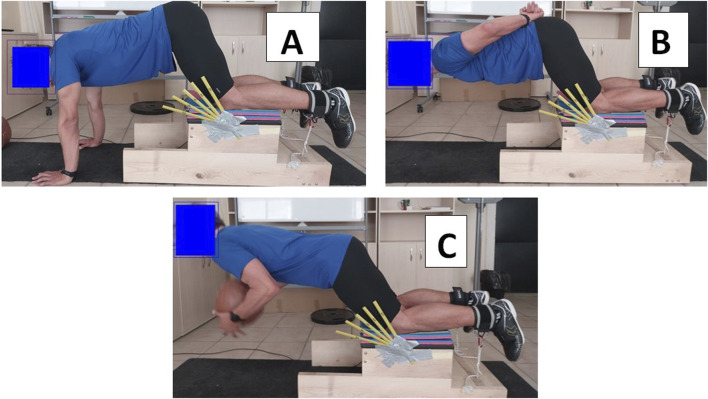
Maximum voluntary isometric contraction at 60^°^ knee flexion **(A)**, 60^°^ knee flexion with body weight **(B)**, and 60^°^ knee flexion with added weight of 5 kg **(C)**.

Three repeated assessments of isometric hamstring strength at 60° knee flexion were performed under different conditions. (1) ISO60 without body weight (ISO60): Participants placed their hands on the floor and their upper bodies in a push-up position (Figure 1A). (2) ISO60 with body weight (ISO60-BW): Participants placed their hands on their hips behind their back (Figure 1B). (3) ISO60 with body weight and additional 5 kg medicine ball (ISO60-BW + AW): Participant held a medicine ball to centre of the xiphoid process (Figure 1C). If the participant was unable to assume this position, one of the examiners held the participant’s body before the test began. All hamstring isometric tests consisted of two 3-s maximal isometric contractions, interspersed with 30-s rest.

**FIGURE 2:**
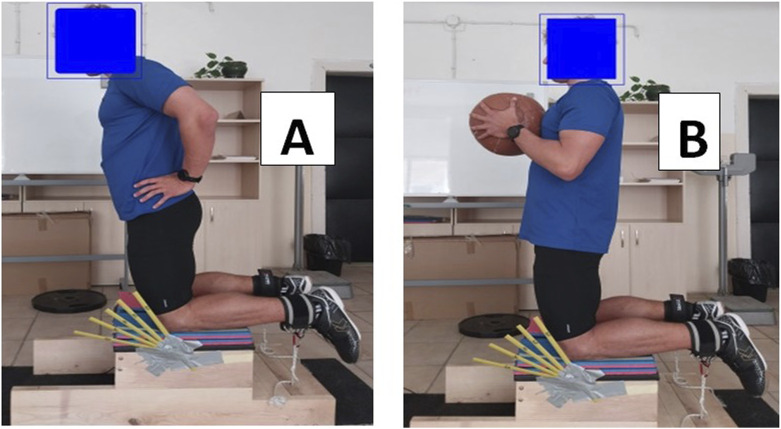
Nordic hamstring exercise with body weight **(A)**, and with additional weight of 5 kg **(B)**.

Maximal eccentric hamstring strength was assessed using body weight and additional weight (holding a 5 kg medicine ball placed in the center of the xiphoid process) (Figures 2A,B. Additionally, the subjects’ ability to resist falling forward during NHE was also evaluated. In the first test, the participant was instructed to gradually lean forward from an initial kneeling position at 90^o^ (180^o^ hip angle) to an angle of 60^o^. In the second test, participants performed NHE with their own weight to achieve either 20^o^ or 45^o^ of knee flexion (Figure 2A). Participants were instructed to lean forward at a constant angular velocity. One investigator was responsible for visually inspecting the final degree to which the participant was able to control himself before falling forward. We used a modified goniometer at various angles (20°, 30°, 40°, 50°, and 60°) to determine the final phase of movement. Rest intervals between trials were separated by 1-min breaks (Drury et al., 2021).

All tests were conducted in a randomized and counterbalanced way to avoid an order effect. Verbal encouragements were given to help the subjects produce maximum effort and focus on the quality of their movements. To minimize possible errors, subjects were familiarized with the measurement and completed a standardized warm-up prior to testing.

### Statistical analysis

The average value (right and left) of peak force (N) across the two repetitions was recorded for further analysis. All data were analyzed using SPSS version 26 (IBM, Armonk, NY). All variables were presented as mean ± standard deviation (SD). The normality of data was calculated using the Shapiro-Wilk test. A one-way ANOVA was implemented to compare the results of three different isometric strength tests. Multiple comparisons were made with *post hoc* Bonferroni correction when ANOVA demonstrated statistical significance. The sphericity was checked using Mauchly’s test, and the significance of F-ratios was adjusted according to the Greenhouse-Geisser or Huynh-Feldt correction. Paired sample t-tests were applied to compare the peak force during ECC60-BW and ECC60-BW + AW when leaning forward from 90^o^ to 60^o^ of knee flexion. An independent sample t-test was used to compare those who were able to achieve the final position at 20^o^ and 45^o^ of knee flexion during NHE. Pearson correlation was used to assess the relationship between peak force during isometric (ISO60, ISO60-BW and ISO60-BW + AW) and eccentric tests (ECC20 and ECC45).

## Results

**FIGURE 3:**
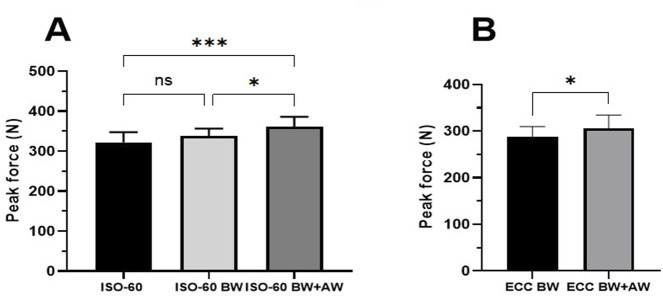
Peak force during maximum isometric hamstring strength test at knee angle of 60° (ISO60), with body weight (ISO60-BW), and added 5 kg extra load (ISO60BW + AW) **(A)**, NHE lean forward from knee flexion of 90°–60° with own weight (ECC60-BW) and added 5 kg extra load (ECC60-BW + AW) **(B)**. Results are presented as mean with 95%CI. ^***^p < 0.000, ^*^p < 0.05, ns p > 0.05.

The result of repeated measures ANOVA showed a significant interaction with a large effect in the three different isometric strength tests (F = 15.14; p = 0.00; ηp2 = 0.41) (Table 1). Post-hoc Bonferroni correction revealed significantly higher force production during maximum voluntary isometric contraction of the hamstrings at 60^o^ knee flexion with body weight + added 5 kg (ISO60-BW + AW) compared to ISO60 with body weight (ISO60-BW) (p = 0.012, 95%CI = 24.49N (4.7–44.2; d = 0.52) and ISO60 (p = 0.000, 95%CI = 42.14N (21.2–62.0); d = 0.76). However, no significant difference was found between ISO60 with body weight (ISO60-BW) and ISO60 (p = 0.083, 95%CI = 17.6N (−1.7–37), d = 0.36) (Figures 3A).

**TABLE 1:** Absolute and relative values of isometric force at 60° knee flexion (ISO60).

Variables	ISO60 (n = 22)	ISO60-BW (n = 22)	ISO60-BW + AW (n = 22)	ISO60-BW + AW vs. ISO60	ISO60-BW + AW vs. ISO60-BW	ISO60-BW vs. ISO60
Absolute peak force (N)	317.64 (56.77)	335.28 (39.23)	359.78 (53.43)	p = 0.000 d = 0.76	p = 0.012 d = 0.52	p = 0.083 d = 0.36
Relative force (N/kg)	4.24 (0.61)	4.50 (0.47)	4.83 (0.67)			
	Sphericity = 0.925, F = 15.14; p = 0.00; ηp2 = 0.41					

**FIGURE 4:**
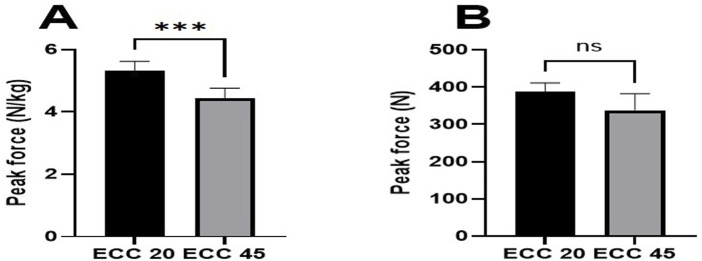
Relative **(A)** and absolute force **(B)** in participants who achieved the final position at 20^°^ and 45^°^ of knee flexion. Results are presented as mean with 95%CI ^*^p < 0.05, ns p > 0.05.

The paired t-test showed significantly higher eccentric peak force during NHE with added weight (ECC60-BW + AW) compared to body weight (ECC60-BW) when leaning forward from 90^o^ to 60^o^ of knee flexion (p = 0.037, 95% CI = 17.94N (1.1–34.6), d = 0.32) (Table 2; Figures 3B). Participants who were able to lean forward up to a knee angle of 20° (ECC20) achieved higher relative force compared to those who reached their critical point at 45° (ECC45) (p = 0.001, 95%CI = 0.88N/kg (0.44–1.32), d = 1.89 (Figures 4A), while there were no significant differences in absolute force production (p = 0.06, 95%CI = 48.79N (−4.11–101.69), d = 0.78 (Figures 4B). In addition, there were no significant differences between eccentric force with added weight (ECC-BW + AW) and eccentric force with own weight (ECC-BW), regardless of the participants’ ability to achieve the final position (p = 0.64, 95%CI = −3.56N (−19.6 to 12.4), d = 0.05).

**TABLE 2:** Absolute and relative values of eccentric force during Nordic hamstring exercise.

Variables	ECC60-BW (n = 22)	ECC60-BW+AW (n = 22)	ECC-BW (n = 22)	ECC-BW+AW (n = 22)	ECC20 (n = 9)	ECC45 (n = 13)
Absolute peak force (N)	281.71 (48.71)	299.66 (62.04)	355.49 (61.64)	351.92 (68.14)	386.71 (32.03)	338.00 (70.20)
Relative force (N/kg)	3.78 (0.61)	4.04 (0.91)	4.75 (0.66)	4.73 (0.91)	5.31 (0.39)	4.43 (0.53)
	p = 0.037 d = 0.32		p = 0.64 d = 0.05		p = 0.06, d = 078 p = 0.001, d = 1.89	

A significant positive correlation was found between peak force during three different isometric tests (ISO60, ISO60-BW and ISO60-BW + AW) and eccentric force during NHE in subjects who were able to lean forward up to 20° of knee flexion (ECC20) (Table 3). The respective r value ranged from 0.70 to 0.76 (p = 0.034–0.016), which indicates large correlations. This resulted in an explained variance of 49%–58% in ECC20. A significant correlation was also revealed between peak force during three different isometric hamstring strength tests and eccentric force when leaning forward to 45° knee angle during NHE. The respective r values ranged from 0.79 to 0.90 (p = 0.001–0.000), indicating large to very large correlations. The explained proportion of variance ranged from 62% to 81% in ECC45.

**TABLE 3:** Pairwise correlations between peak force during isometric and eccentric hamstring strength tests.

Test conditions	ECC20 (n = 9) r-coefficient, p-value	ECC45 (n = 13) r-coefficient, p-value
ISO60 (n = 22)	0.767 (0.41–0.93), p = 0.016	0.901 (0.74–0.97), p = 0.000
ISO60-BW (n = 22)	0.705 (0.19–0.93), p = 0.034	0.787 (0.43–0.96), p = 0.002
ISO60 BW + AW (n = 22)	0.713 (0.39–0.98), p = 0.031	0.817 (0.68–0.97), p = 0.001

## Discussion

The present study examined the effect of body weight and additional weight on isometric and eccentric hamstring strength. Furthermore, the study evaluated a critical point of maximal force production during NHE, where the increasing external load of gravity acting on the upper body exceeds the individual’s maximum eccentric hamstring strength. Maximum isometric force was significantly greater with an additional weight (ISO60-BW + AW) compared to with body weight (ISO60-BW), and without it (ISO60). Similarly, eccentric force production was higher with additional weight (ECC60-BW + AW) than with own weight (ECC60-BW). There was no significant difference between ECC-BW + AW and ECC-BW in eccentric force production, regardless of the participant’s ability to reach the final position at 20° knee flexion. Relative eccentric force differed significantly between participants who achieved a final position of 20^o^ and those who achieved 45^o^ of knee flexion.

While previous work has assessed peak isometric hamstring strength using different knee angle positions (e.g., ISO-prone vs. ISO 30^o^ knee flexion) (Taylor et al., 2025) and (30^o^ vs. 90^o^ knee flexion) (Read et al., 2019), this is the first study to explore isometric hamstring strength with body weight ISO60-BW + AW produces the highest maximum isometric force compared to either ISO60-BW (24.19 N) or ISO60 (42.12 N). Peak isometric force with body weight (ISO60-BW) increased by 4.5 N/kg per 1 kg of body mass at 60° knee flexion. These findings are supported by Connor et al. (2025) who found that torque gradually increases (1.16–2.20 Nm/kg) during isometric Copenhagen adduction test with load increase (105%–140% of body mass) in union rugby players. The improvement of force production during ISO60–BW and ISO60-BW + AW can be attributed to pre-tension before isometric contraction, which results in enhanced activation of muscle groups against the resistance of body weight and added weight. Additionally, isometric exercise with an additional weight involves more than just isolation of the hamstring muscle (BFlh, BFsh, ST, and SM) and the spinal reflex against gravity and external load resistance (Hennemans’s size principle). When adapting isometric training to increase maximum strength, it should be performed at the maximal voluntary contraction (MVC) of 80%–100%, with duration of 1–5 s (total 30–90 s per session) (Lum and Barbosa, 2019). We therefore suggest extending the variation of loads (with body weight and external weight) during isometric exercise to improve hamstring muscle cross-sectional area and strength.

Body mass has been shown to contribute to age-related increase in absolute Nordic hamstring strength (Markovic et al., 2020), with peak force increasing by 4.4 N per 1 kg BW in elite Gaelic football players (Roe et al., 2018). Similarly, Buchheit et al. (2016) found an increase by 4 N per 1 kg of body mass in maximal eccentric knee flexor strength (with predictive equation 4 x BW (kg) + 26.1). Consistent with this, our results showed that ECC-BW increased force by 4.7 N per 1 kg of BW. Moreover, peak eccentric force was greater with an additional 5 kg load (ECC60-BW + AW) than with own weight (ECC60-BW) (17.9 N, 6.4%) during leaning forward from 90° to 60° of knee flexion. A different approach implemented by Amundsen et al. (2023) revealed that eccentric force during NHE performed with added 5 kg was significantly greater in females (+8 N, 2%) and in males (+18 N, 4%) than ECC-BW.

The ability to control the final phase during NHE is one of the most effective methods for determining training intensity, as the load is increased when the athlete can withstand the forward fall for a longer period (Mjølsnes et al., 2004). To ensure a supramaximal NHE, Bourne et al. (2017) added the weight plate from 2.5 kg to 25 kg for participants who could complete the final phase within 10–20°. With respect to this, our results showed that 9 of 22 participants who were able to achieve the final position of 20^o^ of knee flexion demonstrated greater relative force (0.88 N/kg) than those who only reached 45^o^ during the NHE. However, there were no significant differences in absolute force production between the two groups. Therefore, it is necessary to take into account relative strength values when analyzing the data, since, especially in the last phase of the NHE (from approximately 45°–20°), body weight greatly influences the individual’s ability to perform this exercise through the full range of motion. In individuals who achieved a critical point of 45° knee flexion during NHE, maximal eccentric force was highly dependent on their maximal isometric force. Even in those who were able to lean forward by up to a critical point of 20°, their eccentric force was dependent on maximal isometric force, but to a lesser extent. They also need an adequate level of eccentric force to be able to perform the movement throughout the range of motion from 90° to 20° during NHE, especially in the lower position from 45° to 20° of knee angle.

We further investigated differences in eccentric force production between ECC-BW and ECC-BW + AW regardless of participants’ ability to reach the final position at 20^o^ knee flexion throughout the range of movement. Our findings showed that maximal eccentric force did not differ significantly between the two conditions. Pervious study on female football players by Amundsen et al. (2022) showed that high-volume Nordic hamstring training did not differ significantly from low-volume training in maximal eccentric force changes with own weight, 5 kg, and 10 kg. Recent findings suggest that achieving a final position at 20° of knee flexion should not be used as an indicator for adding extra load to maximize eccentric force production (Amundsen et al., 2023). However, we cannot overlook these results, as several of our subjects still encounter limitations when performing NHE.

Foot, knee, and body position during ISO/ECC exercise contributes to the production of maximal force (Ogborn et al., 2021; Taylor et al., 2025). We observed that conducting between ECC20, ECC45 and ISO60 with body weight (ISO60-BW) and with additional load (ISO60-BW + AW) impose similar biomechanical demands at different contraction. Moreover, the body position and knee angle influence participants’ ability against gravity and to resist forward falling during this test. We found a significant correlation between ISO60 knee flexion (i.e., without weight, with body weight, and with additional weight) and NHE. This correlation explained between 62% and 81% of the variance at ECC45 (*R* ^2^ = 0.62–0.81) and between 49% and 58% at ECC20 (*R* ^2^ = 0.49–0.58). Luchner et al. (2021) found a significant correlation between maximum bilateral eccentric (MBHES) and unilateral isometric strength (MUIHS) in Alpine ski racers (r = 0.74–0.84, p = 0.001). This study concluded that MBRHS test is better for determining maximum hamstring force in young Alpine skiers. Given the multi-disciplinary athletic backgrounds of our participants, the integration of isometric and eccentric testing is warranted, as their distinct biomechanical profiles provide complementary information for rehabilitation and sport performance.

The limitation of this study is the small number of participants due to the difficulty of performing ISO60 with their weight and additional weight. Another possible confounding factor was heterogeneity in participants’ training backgrounds across different sports disciplines, which may have influenced their capacity to conduct the ISO/ECC hamstring test. Further research should involve a larger sample of participants already familiar with ISO/ECC hamstring exercises at different protocols (e.g., knee flexion at different angles, body positions, and diverse load interventions) to support these findings. Despite using standardized protocols and a calibrated device, slight inconsistencies in participant effort during maximum-effort ISO/ECC contractions may have caused variability. In addition, the next study should also examine hamstring EMG to observe increase in muscle activation under different conditions (with body and additional weight) and contractions (ISO/ECC).

## Conclusion

Peak force during maximum voluntary isometric hamstring contraction at 60° knee flexion with an added weight of 5 kg is significantly higher compared to the same exercise with body weight (6.5%) and without it (11.6%). However, peak isometric force is not significantly different when this exercise is performed with body weight than without it (4.8%). This means that maximal isometric hamstring strength is influenced by body mass and additional load.

In addition, eccentric hamstring strength during Nordic hamstring exercise when leaning forward from 90° to 60° of knee flexion is significantly greater with an added weight of 5 kg than with own weight (6.4%). However, there is no significant difference between peak eccentric force with body weight and additional weight, regardless of the participant’s ability to reach the final position at 20° of knee flexion. Relative peak force during Nordic hamstring exercise is higher in participants who are able to lean forward up to a 20° of knee flexion than in those who reach their critical point at 45° of knee flexion. However, there are no significant differences in absolute force production during NHE up to 20° and 45° of knee flexion. This demonstrates the importance of determining relative values when assessing eccentric hamstring strength.

Furthermore, peak isometric force at 60° of knee flexion with added extra load, with body weight, and without it is associated with peak eccentric force during NHE when leaning forward up to 45° as well as 20° of knee flexion. These three isometric tests predict 62%–81% of the variance in NHE eccentric force at 45° of knee flexion and 49%–58% at 20° of knee flexion. These findings suggest that isometric and eccentric measures of hamstring strength are interdependent. However, eccentric hamstring strength during NHE is more dependent on maximal hamstring strength when this exercise is performed to a critical point of 45° than 20° of knee flexion. Therefore, we recommend that both tests be integrated into the functional assessment of athletes who extensively use running with changes of direction, including sudden decelerations and accelerations.

## Data Availability

The original contributions presented in the study are included in the article/supplementary material, further inquiries can be directed to the corresponding author.
